# Adaptive Beamforming Applied to OFDM Systems

**DOI:** 10.3390/s18103558

**Published:** 2018-10-20

**Authors:** Tiago F. B. de Sousa, Dalton S. Arantes, Marcelo A. C. Fernandes

**Affiliations:** 1 Department of Computer Engineering and Automation, Federal University of Rio Grande do Norte–UFRN, 59078-970 Natal, RN, Brazil; tiagoair@gmail.com; 2 Department of Communications, School of Electrical and Computer Engineering, State University of Campinas–UNICAMP, 13083-970 Campinas, Brazil; dsarantes@gmail.com

**Keywords:** OFDM, adaptive beamforming, Least Mean Squares (LMS), guard time

## Abstract

This work proposes an adaptive beamforming scheme applied to time domain, pre-FFT (Fast Fourier Transformation), Orthogonal Frequency-Division Multiplexing (OFDM) systems. This scheme improves the performance and the capacity of OFDM systems, using a supervised adaptive algorithm, with frequency domain multiplexed pilots of the OFDM system as a reference. The simplicity of the proposed structure, as well as the method used to obtain reference signals for the adaptive beamforming, are essential aspects that distinguish this paper from other publications. Details on the operation of the proposed scheme, as well as the performance curves, are presented in this manuscript. The proposal investigated here allows a significant reduction in the guard interval of the OFDM system, thereby increasing its robustness or transmission capacity.

## 1. Introduction

In digital communication systems, signals are corrupted by various factors, the most common including white noise and the Intersymbol Interference (ISI) [1]. White noise, which can be characterized as Gaussian random variables, can be minimized with the use of channel encoders [1]. However, the ISI, which is caused by different reflections of transmitted signals, is not efficiently handled by these encoders. The ISI represents the overlapping symbols from the same information source, that is they interfere with each other in the time domain. The ISI limits channel capacity and is one of the greatest problems of the current digital communication systems [1]. However, the receivers can use the spatial filters with the beamforming strategy to reduce the ISI [2,3]. The spatial filters are sensors of antenna arrays, and they can improve a signal from a specific direction with the beamforming strategy.

The OFDM systems have reduced transmission capacity due to the use of the Guard Interval (GI), which in some cases is about 25% of the signal band [4]. On the other hand, the use of a cyclic prefix as GI decreases the ISI, reducing the problem to changes in phase and amplitude that can be minimized with the channel equalizer in the frequency domain [4]. The channel equalizer, which is used on typical OFDM devices, requires adaptive interpolation algorithms associated with frequency domain multiplexed pilot signals. The referenced pilot signals can be distributed, such as the Ultra High Definition Television (UHDTV) with the Integrated Services Digital Broadcasting-Terrestrial (ISDB-T) system [5], Digital Video Broadcasting-Terrestrial (DVB-T), DVB-Second Generation Terrestrial (DVB-T2-Lite) [6], the Brazilian digital television system (Sistema Brasileiro de TV Digital-SBTVD), ISDB-T International (ISDB-T${}_{B}$) [7,8,9], and Long-Term Evolution (LTE-A) [10] or in sequence, as in IEEE 802.11 [11,12] and IEEE 802.15.3c [10,13].

It is essential to observe that a new solution for optimizing the OFDM system also can be applied to the next generation networks based on Single-Carrier Orthogonal Frequency Division Multiple Access (SC-OFDMA). The SC-OFDMA is another approach technique that is based on the OFDM [14,15,16,17,18].

Adaptive beamforming strategies on OFDM receivers have been the focus of many previous papers that addressed different methods. The methods can differ as to the positioning of the gains of the adaptive beamforming that is placed at pre-FFT (Fast Fourier Transformation) or post-FFT [19,20,21,22]. After the FFT, there is the open eye condition in the received symbols; however, for an OFDM beamforming with *K* antennas and *C* subcarriers, the post-FFT scheme needs to execute *K* FFT operations, and the adaptive algorithm needs to update K×C gains. In the pre-FFT project, only two FFT operations and *K* gains’ updating are necessary [19,22]. There are also methods called analog beamforming in which the gains of the array are adjusted by Variable Gain Amplifiers (VGAs) in the Radio Frequency (RF) part [23,24]. The analog beamforming is an attractive technique when the number of antenna elements increases.

In the works presented in [25,26], the MMSEbeamforming algorithm for the pre-FFT OFDM system was applied to a channel assumed to be frequency selective fading. However, the scheme needs the channel estimation to calculate the parameters of the spatial filter. The pre-FFT beamforming based on eigenanalysis and post-FFT subcarrier diversity was proposed in [27,28]. In this case, the eigenvalues and the corresponding eigenvectors were used to determine the parameters of the spatial filter. Similar to the proposal in [25], the work shown in [29] needed the knowledge of the Direction Of Arrival (DOA) and channel fading information. In [30], a method is proposed that uses the Channel State Information (CSI) matrix, and [22] developed a performance evaluation of pre- and post-FFT beamforming. The work in [31] proposed a post-FFT beamformer based on the estimated one-tap channel coefficient associated with a pre-FFT switched-beam device.

It is crucial to observe that there is no open eye condition in the time domain, pre-FFT device part, and this forces most works in the literature not to use the adaptive scheme without prior knowledge of the channel. In other words, there are no reference symbols in the OFDM time domain, and for that, it is necessary for the channel estimation to update the beamforming gains. The works presented in [19,22,32,33,34,35] proposed the alternative method using beamforming with the Least Mean Squares (LMS) and Recursive Least Squares (RLS) adaptive algorithms in the time domain, pre-FFT, without channel estimation. This method updates beamforming gains in the pre-FFT; however, it calculates the error information in the post-FFT, and after, the error signal returns back to the pre-FFT adaptive algorithm. However, for each antenna gain updating, the reference signal (the OFDM pilots) arrives delayed in *C* samples (or one OFDM symbol) from the antenna input signal, and this desynchronization generates convergence problems on the adaptive algorithms. The new approaches using machine learning and optimization algorithms are applied to the adaptive beamforming [36,37]. However, these techniques have a high computational complexity when compared with the LMS algorithm.

This work presents a new implementation of the pre-FFT beamforming, which uses an adaptive spatial filter trained with the LMS algorithm with samples of the OFDM symbol in the time domain. The proposed scheme in this paper does not depend on the channel estimation and uses two operation modes, called here semi-supervised and semi-unsupervised, where the reference signal is an estimated OFDM symbol created in the frequency domain. For the semi-supervised mode, the reference signal is generated from pilot symbols in the frequency domain (or pilot carriers) distributed or in sequence. For the semi-unsupervised mode, the scheme proposed here uses the pilot symbols associated with decided data symbols (or decided data carriers) using the Decision Directed (DD) algorithm. Different from the proposals shown in [19,22,33,34,35], the error signal is calculated in the time domain, and the reference signals of the antenna input signal are not delayed.

## 2. OFDM Communication System and Beamforming

**Figure 1:**
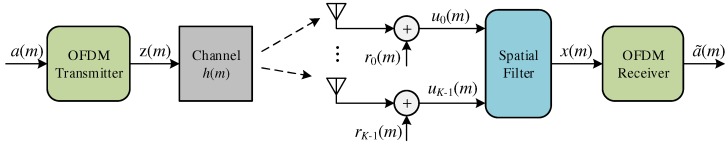
Discrete baseband OFDM system with antenna array.

Figure 1 shows the structure of an OFDM discrete baseband system where the source of information is transmitting symbols a(m). The symbol, a(m), is part of the dataset $A_{M}=a_{0},a_{1},\ldots ,$ $a_{M-1}$ of *M* symbols, and they are transmitted over a period of $t_{s}$ seconds. Each *m*-th symbol represents a word of *B* bits, and $t_{s}$ can be called the sampling period or symbol interval. Table 1 lists the major notations adopted in this paper.

**Figure 2:**
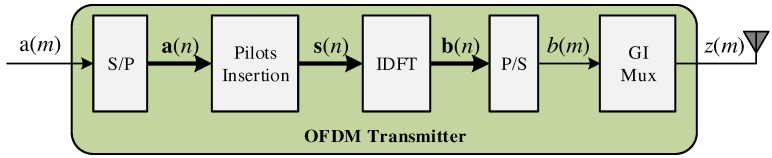
Illustration of the discrete baseband OFDM transmitter. S/P, Serial-to-Parallel converter; IDFT, Inverse Discrete Fourier Transform; P/S, Parallel-to-Serial converter; GI, Guard Interval.

The OFDM transmission technique consists of transforming a Single Carrier (SC) signal, of bandwidth *W* Hz, into a signal formed by *C* carriers of bandwidth W/C Hz. The OFDM scheme parallelizes one data source in several other data sources transmitted in orthogonal sub-carriers [4]. One advantage of this type of transmission is that each sub-carrier may have a bandwidth smaller than the coherence bandwidth of the channel. In other words, each OFDM symbol may have a period longer than the delay spread of the channel, improving the robustness of the system against ISI [4]. The OFDM symbol period, *T*, is given by:

(1) $$T=t_{s}\cdot C,$$

where the symbol interval, $t_{s}$, also coincides with the sampling period of the OFDM symbol. Figure 2 illustrates the OFDM transmitter where the vector, a(n), expressed as:

(2) $$a\left(n\right)=\left[\begin{array}{c} a_{0}\left(n\right)\\ \vdots \\ a_{p}\left(n\right)\\ \vdots \\ a_{C_{D}-1}\left(n\right) \end{array}\right]=\left[\begin{array}{c} a(m+nC_{D})\\ \vdots \\ a(m-p+nC_{D})\\ \vdots \\ a(m-C_{D}+1+nC_{D}) \end{array}\right],$$

collects $C_{D}$ symbols, a(m), after the Serial-to-Parallel converter (S/P). In the OFDM transmitter scheme, the signal $a_{p}\left(n\right)$ represents the *p*-th data carrier associated with the *n*-th OFDM symbol in the frequency domain.

The data carriers are multiplexed with $C_{P}$ pilot carriers, o(n), expressed as:

(3) $$o\left(n\right)=\left[\begin{array}{c} o_{0}\left(n\right)\\ \vdots \\ o_{k}\left(n\right)\\ \vdots \\ o_{C_{P}-1}\left(n\right) \end{array}\right],$$

where $o_{k}\left(n\right)$ is the *k*-th pilot carrier in the *n*-th OFDM symbol. After pilot insertion are inserted $C_{ZP}$ zeros (zero padding) generating a set of $C=C_{D}+C_{P}+C_{ZP}$ carriers, characterized by:

(4) $$s\left(n\right)=\left[\begin{array}{c} s_{0}\left(n\right)\\ \vdots \\ s_{j}\left(n\right)\\ \vdots \\ s_{C-1}\left(n\right) \end{array}\right]=\left[\begin{array}{c} s(m+nC)\\ \vdots \\ s(m-j+nC)\\ \vdots \\ s(m-C+1+nC) \end{array}\right]$$

where,

(5) $$s_{j}\left(n\right)=\left\{\begin{array}{cc} o_{k}\left(n\right), & \mathrm{if}\;(j\in \mathrm{pilot}\;\mathrm{carrier}\;\mathrm{position})\\ a_{p}\left(n\right), & \mathrm{if}\;(j\in \mathrm{data}\;\mathrm{carrier}\;\mathrm{position})\\ 0, & \mathrm{if}\;(j\notin \mathrm{pilot}\;\mathrm{or}\;\mathrm{data}\;\mathrm{carrier}\;\mathrm{position}) \end{array}\right..$$

The vector, s(n), is then processed by the IDFT generating a new *n*-th OFDM symbol, b(n), characterized as:

(6) b(n)=IDFT(s(n)),

where:

(7) $$b\left(n\right)=\left[\begin{array}{c} b_{0}\left(n\right)\\ \vdots \\ b_{j}\left(n\right)\\ \vdots \\ b_{C-1}\left(n\right) \end{array}\right]=\left[\begin{array}{c} b(m+nC)\\ \vdots \\ b(m-j+nC)\\ \vdots \\ b(m-C+1+nC) \end{array}\right],$$

the signal, $b_{j}\left(n\right)$, is the *j*-th sample of the *n*-th OFDM symbol. The GI (see the GI mux block in Figure 2) insertion in a vector representation can be characterized by:

(8) $$\mathrm{GI}\left(n\right)=\left[\begin{array}{c} GI(n-C)\\ \vdots \\ GI(n-C-N_{GI}+1) \end{array}\right],$$

where $N_{GI}$ is the number of the GI samples. The combination of Matrices 7 and 8 gives:

(9) $$z\left(n\right)=\left[\begin{array}{c} b(n)\\ ----\\ \mathrm{GI}(n) \end{array}\right],$$

where z(n) is the OFDM symbol composed of $C+N_{GI}$ samples. Usually, the size of GI is expressed as a fraction of the data carriers, $C_{D}$, where $GI=1/(C_{D}/N_{GI})$.

The symbols are transmitted (by the OFDM transmitter) through a channel, h(m), subject to the ISI and additive white Gaussian noise (AWGN), r(m). The channel impulse response, h(m), can be expressed as:

(10) $$h\left(m\right)=\sum_{i=0}^{L-1}\alpha_{i}\delta \left(m-\tau_{i}\right),$$

where *L* is the number of paths of the channel, $\alpha_{i}$ is the complex gain of the *i*-th path and $\tau_{i}$ is an integer value representing the delay of the *i*-th path at time *m*. The receiver, shown in Figure 1, processes the signal u(m), resulting from the channel and expressed by:

(11) $$u\left(m\right)=\sum_{i=0}^{L-1}\alpha_{i}z\left(m-\tau_{i}\right),$$

where z(m) is the symbol at the output of the OFDM transmitter or the sample of the OFDM symbol. Equation (11) can also be written in vector form, as follows:

(12) $$u\left(m\right)=h^{T}z\left(m\right),$$

where h(m) is the vector of complex channel gains, with ISI, length *L*, given by:

(13) $$h=\left[\begin{array}{c} \alpha_{0}\\ \vdots \\ \alpha_{L-1} \end{array}\right],$$

and z(m) is the vector of channel delays applied to the transmitted signal z(m), given by:

(14) $$z\left(m\right)=\left[\begin{array}{c} z(m-\tau_{0})\\ \vdots \\ z(m-\tau_{L-1}) \end{array}\right].$$

Rewriting Equation (12) for the *n*-th OFDM symbol and assuming that $N_{GI}\geq L$, the $C+N_{GI}$ samples of the received OFDM symbol are given by:

(15) $$u\left(n\right)=Z^{T}\left(n\right)h,$$

where Z(n) is the delay matrix formed by the vectors shown in Equation (24), which can be represented by:

(16) $$Z\left(n\right)=\left[\begin{array}{ccccc} B^{\prime }\left(n\right) & | & B^{\prime \prime }\left(n\right) & | & \mathrm{GI}(n) \end{array}\right],$$

where $B^{\prime }\left(n\right)$ is the ISI portion within the OFDM symbol characterized as:

(17) $$B^{\prime }\left(n\right)=\left[\begin{array}{ccc} b(m-d_{0,0}) & \cdots & b(m-d_{0,C-L-1})\\ \vdots & \ddots & \vdots \\ b(m-d_{L-1,0}) & \cdots & b(m-d_{L-1,C-L-1}) \end{array}\right],$$

whereas $B^{\prime \prime }\left(n\right)$ is the ISI portion between the OFDM and GI samples, being described as:

(18) $$B^{\prime \prime }\left(n\right)=\left[\begin{array}{ccc} b(m-d_{0,C-L}) & \cdots & b(m-d_{0,C-1})\\ \vdots & \ddots & \vdots \\ GI(m-d_{0,C-L}) & \cdots & GI(m-d_{L-1,C-1}) \end{array}\right],$$

and the GI(n) matrix describes the interference within the GI. In the $B^{\prime }\left(n\right)$ and $B^{\prime \prime }\left(n\right)$ matrices, the $d_{i,j}$ variable is characterized as:

(19) $$d_{i,j}=\tau_{i}-j.$$

**Figure 3:**
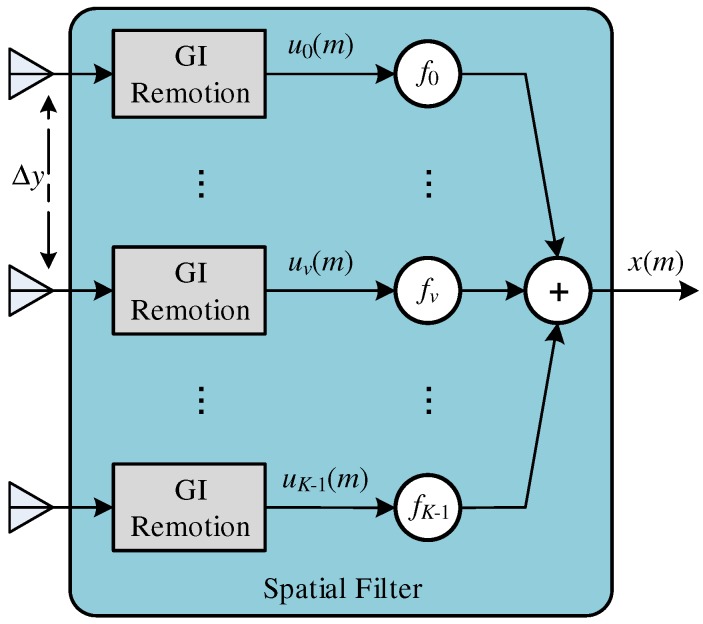
Antenna array with *K* equally-spaced sensors.

Figure 3 shows a Linearly Equally-Spaced (LES) array where the antenna elements are arranged along the *y*-axis, with spacing of Δy. It is assumed that all the multipath arrives at the array in the vertical plane, with the Angle Of Arrival (AOA) of θ radians with respect to the to the *x*-axis orthogonal to the *y*-axis. Each *v*-th element of the antenna array is weighted by a complex gain, $f_{v}$, and the spacing Δy should generally be greater than or equal to λ/2.

The signal, u(m), received by the *v*-th antenna element is expressed as:

(20) $$u_{v}\left(m\right)=\sum_{i=0}^{L-1}\alpha_{i}z\left(m-\tau_{i}\right)e^{-j\left(v\beta \right)\Delta y\cos\left(\theta_{i}\right)}+r_{v}\left(m\right),$$

where β=(2π)/λ, $\theta_{i}$ is the AOA associated with the *i*-th channel path and $r_{v}\left(m\right)$ is the noise associated with each *v*-th antenna element. Rewriting the Equation (20) based on Equation (15) gives:

(21) $$u_{v}\left(n\right)=B^{T}\left(n\right)O_{v}\left(\theta \right)h+r_{v}\left(n\right)$$

where B(n) represents the signal after GI remotion, GI(n) (see Figure 3 and Equation (16)), that is:

(22) $$B\left(n\right)=\left[\begin{array}{ccc} B^{\prime }\left(n\right) & | & B^{\prime \prime }\left(n\right) \end{array}\right]$$

and $O_{v}\left(\theta \right)$ is a diagonal matrix expressed as:

(23) $$O_{v}\left(\theta \right)=\left[\begin{array}{ccc} e^{-j\left(v\beta \right)\Delta y\cos\left(\theta_{0}\right)} & \cdots & 0\\ \vdots & \ddots & \vdots \\ 0 & \cdots & e^{-j\left(v\beta \right)\Delta y\cos\left(\theta_{L-1}\right)} \end{array}\right]$$

and:

(24) $$\theta =\left[\begin{array}{c} \theta_{0}\\ \vdots \\ \theta_{L-1} \end{array}\right].$$

**Figure 4:**
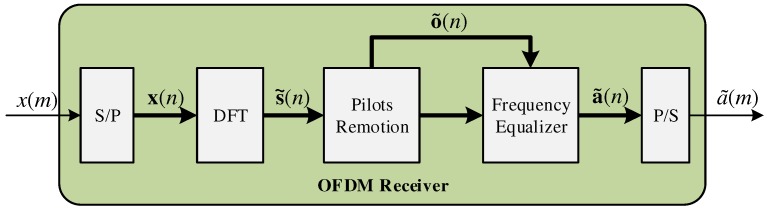
Illustration of the discrete baseband OFDM receiver. DFT, Discrete Fourier Transform.

The combined output of the signals of the *K* antenna elements, x(m), is represented by:

(25) $$x\left(m\right)=\sum_{v=0}^{K-1}f_{v}u_{v}\left(m\right)=\sum_{v=0}^{K-1}\sum_{i=0}^{L-1}f_{v}\alpha_{i}z\left(m-\tau_{i}\right)e^{-j\beta v\Delta x\cos(\theta_{i})}+f_{v}r_{v}\left(m\right).$$

where using Equation (21), the OFDM symbol received, after the LES, can be shown as:

(26) $$x\left(n\right)=\sum_{v=0}^{K-1}f_{v}u_{v}\left(n\right)=\sum_{v=0}^{K-1}f_{v}B^{T}\left(n\right)O_{v}\left(\theta \right)h+\sum_{v=0}^{K-1}f_{v}r_{v}\left(n\right)=B^{T}\left(n\right)A\left(\theta \right)h+\sum_{v=0}^{K-1}f_{v}r_{v}\left(n\right)$$

where:

(27) $$A\left(\theta \right)=\sum_{v=0}^{K-1}f_{v}O_{v}\left(\theta \right)$$

and x(n) is expressed as:

(28) $$x\left(n\right)=\left[\begin{array}{c} x_{0}\left(n\right)\\ \vdots \\ x_{p}\left(n\right)\\ \vdots \\ x_{C-1}\left(n\right) \end{array}\right]=\left[\begin{array}{c} x(m+nC)\\ \vdots \\ x(m-p+nC)\\ \vdots \\ x(m-C+1+nC) \end{array}\right],$$

where $x_{p}\left(n\right)$ is the *p*-th sample of the *n*-th OFDM symbol. The variable A(θ) is the array factor, and it determines the antenna pattern and the direction gain. Adjusting the *K* antenna array gains, $f_{v}$ (v=0,⋯,K−1), it is possible to select any direction for maximum gain [2]. After the spatial filter, the signal, x(n), is processed by the OFDM receiver, shown in details in Figure 4.

The estimated carriers associated with the *n*-th OFDM symbol is given by:

(29) $$\tilde{s}\left(n\right)=\mathrm{DFT}\left(x\left(n\right)\right)=\mathrm{DFT}\left(B^{T}\left(n\right)A\left(\theta \right)h\right)+\mathrm{DFT}\left(\sum_{v=0}^{K-1}f_{v}r_{v}\left(n\right)\right)=s\left(n\right)c^{T}I+g\left(n\right)$$

where c can be expressed as:

(30) $$c\equiv \mathrm{DFT}\left(\left[\begin{array}{c} \gamma \\ ----\\ 0 \end{array}\right]\right)=\mathrm{DFT}\left(\left[\begin{array}{c} A(\theta )h\\ ----\\ 0 \end{array}\right]\right).$$

The vector γ is a “better version” of the channel, h, and it is given by:

(31) $$\gamma =\left[\begin{array}{c} \gamma_{0}\\ \vdots \\ \gamma_{D-1} \end{array}\right],$$

where D≤L and 0 is a vector with C−D zeros. The vector g(n) is the OFDM symbol noise in the frequency domain, and it is indicated as:

(32) $$g\left(n\right)=\mathrm{DFT}\left(\sum_{v=0}^{K-1}f_{v}r_{v}\left(n\right)\right)=\left[\begin{array}{c} g_{0}\left(n\right)\\ \vdots \\ g_{C-1}\left(n\right) \end{array}\right].$$

The frequency equalizer (see Figure 4) compensates the channel effects, c, using received pilot carries, $\tilde{o}\left(n\right)$, expressed as:

(33) $$\tilde{o}\left(n\right)=\left[\begin{array}{c} {\tilde{o}}_{0}\left(n\right)\\ \vdots \\ {\tilde{o}}_{C_{P}-1}\left(n\right) \end{array}\right]=o\left(n\right)c^{T}I+g_{P}\left(n\right)$$

where $g_{P}\left(n\right)$ is the pilot noise. There are several techniques and schemes for the OFDM frequency equalizer, and the conventional strategy uses the interpolation algorithm with pilot carriers to create a channel estimation [4]. With the channel information, it is possible to compensate the channel effects and estimate the transmitted data carriers, $\tilde{a}\left(n\right)$.

Equations (29) and (30) show that the beamforming scheme can minimize the ISI problem by reducing the attenuation (A(θ)h) and length (D≤L) of the channel, which helps the frequency domain equalizer and also enables the reduction of the GI size, $N_{GI}<L$. Another important point is that the beamforming is the Single Input Multiple Output (SIMO) system, and the pilot carriers work with the Signal-to-Noise Ratio (SNR) over the SIMO system; this enables the reduction of the noise enhancement in the frequency equalizer [38]. Thus, the question is how to efficiently find the spatial filters’ gains,

(34) $$f=\left[\begin{array}{c} f_{0}\\ \vdots \\ f_{K-1} \end{array}\right],$$

that allow these improvements.

## 3. Proposed Method for OFDM Adaptive Antenna Array

**Figure 5:**
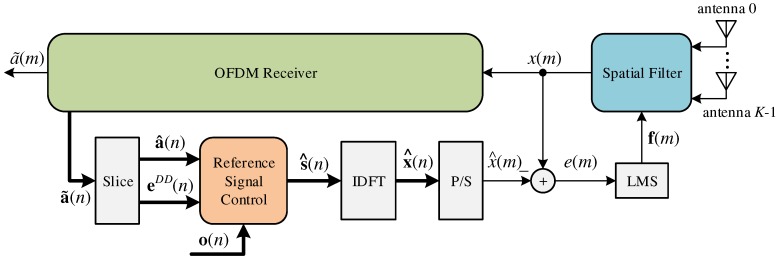
Adaptive antenna array proposed for the OFDM system.

Figure 5 shows the adaptive beamforming proposed in this work, where the *K* spatial filter gains, f(m), are adjusted in each *m*-th sample of the OFDM symbol using the LMS algorithm. The error signal, e(m), is calculated in time domain and is given as:

(35) $$e\left(m\right)=\hat{x}\left(m\right)-x\left(m\right)$$

where $\hat{x}\left(m\right)$ is part of the vector $\hat{x}\left(n\right)$ expressed as:

(36) $$\hat{x}\left(n\right)=\left[\begin{array}{c} {\hat{x}}_{0}\left(n\right)\\ \vdots \\ {\hat{x}}_{C-1}\left(n\right) \end{array}\right]=\mathrm{IDFT}\left(\hat{s}\left(n\right)\right).$$

The reference vector, $\hat{s}\left(n\right)$, is created in the frequency domain by the module called Reference Signal Control (RSC) (see in Figure 5) and is expressed as:

(37) $$\hat{s}\left(n\right)=\left[\begin{array}{c} {\hat{s}}_{0}\left(n\right)\\ \vdots \\ {\hat{s}}_{j}\left(n\right)\\ \vdots \\ {\hat{s}}_{C-1}\left(n\right) \end{array}\right]=\left[\begin{array}{c} \hat{s}\left(m+nC\right)\\ \vdots \\ \hat{s}\left(m-j+nC\right)\\ \vdots \\ \hat{s}\left(m-C+1+nC\right) \end{array}\right]$$

where,

(38) $${\hat{s}}_{j}\left(n\right)=\left\{\begin{array}{c} o_{k}\left(n\right),\;\;\mathrm{if}\;\left(j\in \mathrm{pilot}\;\mathrm{carrier}\;\mathrm{position}\right)\\ 0,\;\;\;\;\;\;\;\;\;\;\;\;\;\mathrm{if}\;\left(j\in \mathrm{data}\;\mathrm{carrier}\;\mathrm{position}\right)\;\mathrm{and}\;(e_{p}^{DD}\left(n\right)\geq E^{DD}\;\mathrm{or}\;n\leq N^{DD})\\ {\hat{a}}_{p}\left(n\right),\;\;\mathrm{if}\;\left(j\in \mathrm{data}\;\mathrm{carrier}\;\mathrm{position}\right)\;\mathrm{and}\;\left(e_{p}^{DD}\left(n\right)<E^{DD}\;\mathrm{or}\;n>N^{DD}\right)\\ 0,\;\;\;\;\;\;\;\;\;\;\;\;\;\mathrm{if}\;(j\notin \mathrm{pilot}\;\mathrm{or}\;\mathrm{data}\;\mathrm{carrier}\;\mathrm{position},\;\mathrm{zero}\;\mathrm{padding}) \end{array}\right.$$

where ${\hat{o}}_{k}\left(n\right)$ is the *p*-th pilot carrier, ${\hat{a}}_{p}\left(n\right)$ is the *p*-th decided data carrier (or decided symbol), the signal $e_{p}^{DD}\left(n\right)$ is the *p*-th direct decision error expressed as:

(39) $$e_{p}^{DD}\left(n\right)={\hat{a}}_{p}\left(n\right)-{\tilde{a}}_{p}\left(n\right)=\mathrm{slice}\left\{{\tilde{a}}_{p}\left(n\right)\right\}-{\tilde{a}}_{p}\left(n\right)$$

where ${\tilde{a}}_{p}\left(n\right)$ is the *p*-th estimated data carrier (or estimated symbol) and the $E^{DD}$ and $N^{DD}$ are parameters managed by RSC. The RSC has three inputs: the pilot carriers, o(n), the decided data carriers, $\hat{a}\left(n\right)$, given by:

(40) $$\hat{a}\left(n\right)=\left[\begin{array}{c} {\hat{a}}_{0}\left(n\right)\\ \vdots \\ {\hat{a}}_{C_{D}-1}\left(n\right) \end{array}\right]=\left[\begin{array}{c} \mathrm{slice}\left\{{\tilde{a}}_{0}\left(n\right)\right\}\\ \vdots \\ \mathrm{slice}\left\{{\tilde{a}}_{C_{D}-1}\left(n\right)\right\} \end{array}\right]=\left[\begin{array}{c} \mathrm{slice}\left\{\tilde{a}\left(m+nC_{D}\right)\right\}\\ \vdots \\ \mathrm{slice}\left\{\tilde{a}\left(m-C+1+nC_{D}\right)\right\} \end{array}\right]$$

and the direct decisions errors, $e_{p}^{DD}\left(n\right)$, expressed as:

(41) $$e^{DD}\left(n\right)=\left[\begin{array}{c} e_{0}^{DD}\left(n\right)\\ \vdots \\ e_{C_{D}-1}^{DD}\left(n\right) \end{array}\right].$$

The RSC uses the rules shown in Equation (38), and it makes a decision if the *p*-th decided data carrier (or data symbol), ${\hat{a}}_{p}\left(n\right)$, will contribute with the reference signal, $\hat{s}\left(n\right)$. The decision is based on two parameters, $E^{DD}$ and $N^{DD}$. The parameter $E^{DD}$ is the upper bound of the direct decision error, and it decides if the *p*-th data carrier (or data symbol), ${\hat{a}}_{p}\left(n\right)$, has an appropriate value to use in the reference signal, $\hat{s}\left(n\right)$, and the parameter $N^{DD}$ controls the time (from that OFDM symbol) the data carriers can start to contribute to the reference signal $\hat{s}\left(n\right)$. Differently than the works presented in the literature, the RSC allows the semi-unsupervised mode, where the reference vector $\hat{s}\left(n\right)$ combines pilot carriers with decided data carriers using the Decision Directed (DD) algorithm.

The LMS algorithm is executed in the OFDM sample time, $t_{s}$ (see Equation (1)), and this enables one to adjust the antenna array gains, f(m), faster than the works proposed in [19,22,32,33,34,35]. The LMS implements the equation given as:

(42) f(m)=f(m−1)+ηe(m)u(m)

where u(m) (see Figure 3) is expressed as:

(43) $$u\left(m\right)=\left[\begin{array}{c} u_{0}\left(m\right)\\ \vdots \\ u_{v}\left(m\right)\\ \vdots \\ u_{K-1}\left(m\right) \end{array}\right]$$

and η is the adaptation step.

It is important to observe that when $n\leq N^{DD}\;\left(n=0,\cdots ,N^{DD}\right)$, the reference signal, $\hat{s}\left(n\right)$, uses just pilot carriers and zeros, and it creates a estimated version of the reference OFDM symbol, $\hat{s}\left(n\right)$. Differently than the works proposed in [19,22,32,33,34,35], the reference signal, ${\hat{s}}_{j}\left(n\right)$, does not need to wait for the *C* OFDM samples (or one OFDM symbol) to update (see Equation (42)).

## 4. Simulations and Results

To validate the adaptive beamforming scheme proposed here, several simulations were performed with the OFDM system. Table 2 shows the constant parameters used in all simulations where they were based on the SBTVD standard [8]. Three channel scenarios (Channel 1, Channel 2 and Channel 3) were simulated according to the parameters presented in Table 3. For each channel, the Bit Error Rate (BER) curves were created for distinct values of the number of antennas (K=1, K=4, K=6, K=8 and K=10) and GI size (GI=1/2 and GI=1/512). Channel coding was not used in any simulation.

**Figure 6:**
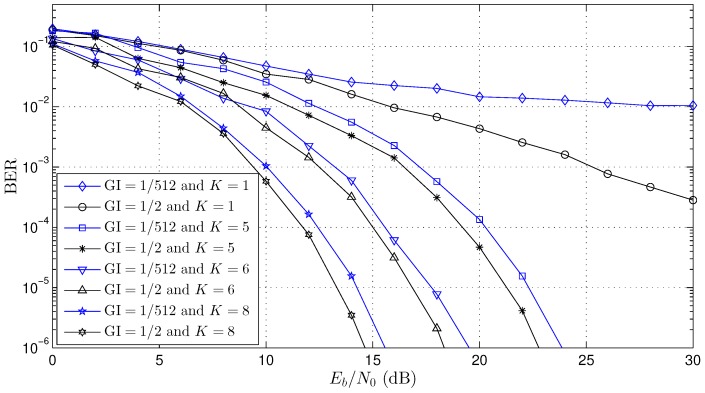
BER performance curve for Channel 1 (see Table 3).

Figure 6 shows the BER performance curve for Channel 1 (see Table 3). Channel 1 is a long channel, L≈104 OFDM samples (see Equations (10) and (11)), that is 12.7 $\mu s/t_{s}\approx 104$. Based on Equations (16)–(18), the minimum number of GI samples, $N_{GI}$, may be greater than 104 ($N_{GI}\geq 104$) in order to avoid the OFDM symbol interference. Regarding the study of the improvements associated with the adaptive beamforming, GI=1/512 ($N_{GI}=4$) and GI=1/2 ($N_{GI}=740$) were used for three numbers of antennas, K=5, K=6 and K=8. The results show that the adaptive beamforming proposal had an excellent performance where, when the $BER=10^{-4}$, the gain was about 10dB, 15dB and 17.5dB for K=5, K=6 and K=8, respectively; even for the cases where $N_{GI}<104$ (GI=1/512), which enables one to work with more bandwidth efficiency. It is essential to observe that there is a small gain, about 2dB, between GI=1/2 and GI=1/512 for all beamforming results (K=5, K=6 and K=8), and this proves the shortest of the channel presented by Equations (29)–(31).

**Figure 7:**
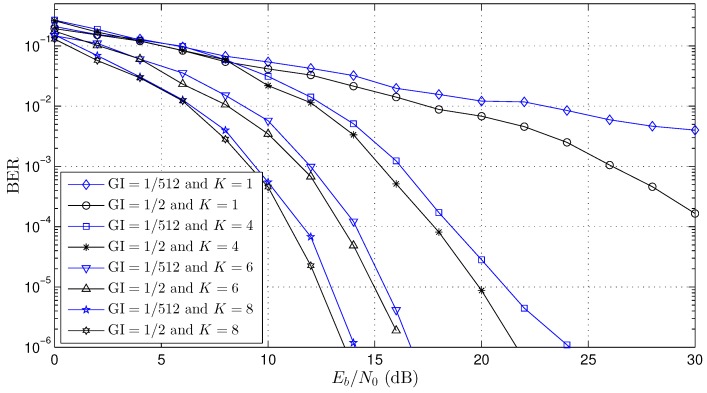
BER performance curve for Channel 2 (see Table 3).

The BER performance curve for Channel 2 (see Table 3) is presented in Figure 7 where it is a typical wireless network channel. The length of the channel *L* is ≈23 OFDM samples (see Equations (10) and (11)), that is 2.8 $\mu s/t_{s}\approx 23$. For this case, the minimal number of the GI samples, $N_{GI}$, may be greater than 23 ($N_{GI}\geq 23$) to avoid the OFDM symbol interference (see Equations (16)–(18)). Similar to the Channel 1 simulations, GI=1/512 ($N_{GI}=4$) and GI=1/2 ($N_{GI}=740$) were used for three numbers of antennas, K=4, K=6 and K=8. The results also show improved performance when compared to the OFDM system (K=1). Using the value of $BER=10^{-4}$, the gain was about 12dB, 17dB and 18.5dB for the K=4, K=6 and K=8, respectively. For cases with $N_{GI}<23$ (GI=1/512), the adaptive beamforming worked, and similar to Channel 1, there is a small gain, <2 dB, between GI=1/2 and GI=1/512 for all beamforming results (K=4, K=6 and K=8).

**Figure 8:**
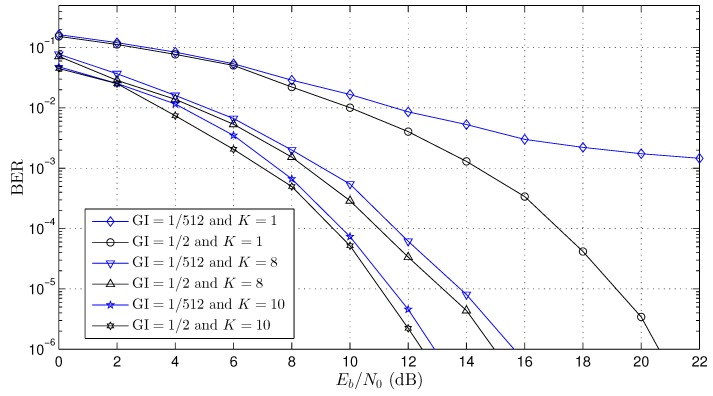
BER performance curve for Channel 3 (see Table 3).

Finally, Figure 8 shows the BER performance curve for Channel 3 (see Table 3). Channel 3 has L≈17 OFDM samples (see Equations (10) and (11)), that is 2.1 $\mu s/t_{s}\approx 17$. Based on Equations (16)–(18), the minimum number of GI samples, $N_{GI}$, may be greater than 17 ($N_{GI}\geq 17$) to not allow the OFDM symbol interference. The results were obtained for two numbers of antennas K=8 and K=10, and the gains (using $BER=10^{-4}$ as reference) were about 6dB and 7.5dB for K=8 and K=10, respectively. Such as other cases, the beamforming worked even for the cases where $N_{GI}<17$ (GI=1/512).

The works presented in [19,22,32,33,34,35] had results with a guard interval great than channel length $N_{GI}>L$, and this is mandatory for solutions where the array beamforming is updated in each OFDM symbol in the time domain. In this work, the array beamforming proposed was updated in each *m*-th sample in the frequency domain, and this allows one to work with a guard interval smaller than channel length $N_{GI}<L$. The BER curves presented in the works [19,22,35] used the OFDM system with K=8 antennas, C=64 carriers ($C_{D}=56$ and $C_{P}=8$) and GI=1/4 ($N_{GI}=16$) in the three-path channel (L=3), that is $N_{GI}>L$. However, in all simulations presented in this work (see Figure 6, Figure 7 and Figure 8), the size of the guard interval ($N_{GI}$) had little influence on the BER performance (<1 dB), and this is a significant contribution when compared with other works presented in the literature. It is important to highlight that the reduction of the GI improves the channel capacity of the OFDM systems.

## 5. Conclusions

This paper proposed a novel beamforming scheme for OFDM systems, with the pre-FFT adaptive algorithm. This adaptive beamforming allows a very low guard interval of the OFDM system, and it enables one to work with more bandwidth efficiency. This strategy used a simple, fast and efficient solution for the OFDM receiver compared to pre-FFT structures found in the literature. The results suggest the feasibility of implementing the proposed scheme in different OFDM receivers, such as for digital TV and wireless LANs standards. Another important aspect concerns the simplicity of the adaptive structure, which does not change the conventional algorithms, reinforcing the feasibility of the proposed scheme.

## Figures and Tables

**Table 1:** Major notations used in this paper.

Notation	Description
GI	The guard interval
*C*	Number of the carriers
$C_{D}$	Number of the data carriers
$C_{P}$	Number of the pilot carriers
$C_{ZP}$	Zero padding size
*T*	OFDM symbol period
$t_{s}$	The sampling period of the OFDM symbol
$N_{GI}$	The number of the GI samples
*L*	The number of paths of the channel
$\alpha_{i}$	The complex gain of the *i*-th path
$\tau_{i}$	The delay of the *i*-th path
*K*	The number of the antenna elements
Δy	Space between the antenna elements

**Table 2:** Constant parameters used in all simulations.

Space between the Antenna Elements (Δy)	λ/2
Sampling period of the OFDM symbol ($t_{s}$)	0.1230 μs
Number of the data carriers ($C_{D}$)	1248 carriers
Number of the pilot carriers ($C_{P}$)	156 carriers
Zero padding size ($C_{ZP}$)	644 carriers
Number of the carriers (*C*)	2048 carriers
OFDM symbol period (*T*)	251.9040 μs
Modulation	16-QAM
Pilots	Spread (SBTVDstandard [8])
Channel coding	none

**Table 3:** Simulated channels.

Channel 1	μs	0.00	0.30	3.50	4.40	9.50	12.70
	dB	0.00	−12.00	−4.00	−7.00	−15.00	−22.00
	AOA	$30^{\circ }$	$50^{\circ }$	$90^{\circ }$	$120^{\circ }$	$150^{\circ }$	$170^{\circ }$
Channel 2	μs	0.00	0.15	0.42	1.51	2.32	2.80
	dB	0.00	−3.09	−6.02	−10.45	−20.00	−26.02
	AOA	$30^{\circ }$	$50^{\circ }$	$90^{\circ }$	$120^{\circ }$	$150^{\circ }$	$170^{\circ }$
Channel 3	μs	0.00	0.74	1.11	1.48	1.85	2.10
	dB	0.00	−20.00	−20.00	−10.00	−14.00	−18.00
	AOA	$30^{\circ }$	$50^{\circ }$	$90^{\circ }$	$120^{\circ }$	$150^{\circ }$	$170^{\circ }$

## References

[1] Proakis J. (2000). Digital Communications.

[2] Liberti J.C., Rappaport T.S. (1990). Smart Antennas for Wireless Communications: IS-95 and Third Generation CDMA Applications.

[3] Haykin S. (2001). Adaptive Filter Theory.

[4] Hanzo L., Munster M., Choi B., Keller T. (2003). OFDM and MC-CDMA for Broadband Multi-User Communications, WLANs and Broadcasting.

[5] Sato A., Shitomi T., Takeuchi T., Okano M., Tsuchida K. Transmission performance evaluation of LDPC coded OFDM over actual propagation channels in urban area. Examination for next-generation ISDB-T. Proceedings of the 2017 IEEE International Symposium on Broadband Multimedia Systems and Broadcasting (BMSB).

[6] Polak L., Kratochvil T. Measurement and evaluation of IQ-Imbalances in DVB-T and DVB-T2-Lite OFDM modulators. Proceedings of the 2017 40th International Conference on Telecommunications and Signal Processing (TSP).

[7] International Telecommunication Union (1999). Channel Coding, Frame Structure and Modulation Scheme for Terrestrial Integrated Services Digital Broadcasting (ISDB-T).

[8] Norma Brasileira ABNT NBR 15601 (2008). Televisão Digital Terrestre–Sistema de Transmissão.

[9] Almeida J.J.H., Lopes P., Akamine C., Omar N. (2018). An Application of Neural Networks to Channel Estimation of the ISDB-TB FBMC System. arXiv.

[10] Luvisotto M., Pang Z., Dzung D. (2017). Ultra High Performance Wireless Control for Critical Applications: Challenges and Directions. IEEE Trans. Ind. Inf..

[11] Glisic S.G. (2006). Advanced Wireless Networks: 4G Technologies..

[12] Bloessl B., Klingler F., Missbrenner F., Sommer C. A systematic study on the impact of noise and OFDM interference on IEEE 802.11 p. Proceedings of the 2017 IEEE Vehicular Networking Conference (VNC).

[13] Liu W.C., Wei T.C., Huang Y.S., Chan C.D., Jou S.J. (2015). All-Digital Synchronization for SC/OFDM Mode of IEEE 802.15. 3c and IEEE 802.11 ad. IEEE Trans. Circuits Syst..

[14] Tsiropoulou E.E., Kapoukakis A., Papavassiliou S. (2016). Uplink resource allocation in SC-FDMA wireless networks: A survey and taxonomy. Comput. Netw..

[15] Myung H.G., Lim J., Goodman D.J. (2006). Single carrier FDMA for uplink wireless transmission. IEEE Veh. Technol. Mag..

[16] Tsiropoulou E.E., Kapoukakis A., Papavassiliou S. Energy-efficient subcarrier allocation in SC-FDMA wireless networks based on multilateral model of bargaining. Proceedings of the 2013 IFIP Networking Conference.

[17] Myung H.G., Goodman D.J. (2008). Single Carrier FDMA: A New Air Interface for Long Term Evolution.

[18] Myung H.G. Introduction to single carrier FDMA. Proceedings of the EUSIPCO.

[19] Seydnejad S., Akhzari S. A combined time-frequency domain beamforming method for OFDM systems. Proceedings of the 2010 International ITG Workshop on Smart Antennas (WSA).

[20] Alihemmati R., Jedari E., Enayati A., Shishegar A.A., Roozbahani M., Dadashzadeh G. Performance of the Pre/Post-FFT Smart Antenna Methods for OFDM-Based Wireless LANs in an Indoor Channel with Interference. Proceedings of the 2006 ICC ’06 IEEE International Conference on Communications.

[21] Pham D.H., Gao J., Tabata T., Asato H., Hori S., Wada T. (2008). Implementation of Joint Pre-FFT Adaptive Array Antenna and Post-FFT Space Diversity Combining for Mobile ISDB-T Receiver. IEICE Trans..

[22] Seydnejad S.R., Akhzari S. (2016). Performance evaluation of pre-and post-FFT beamforming methods in pilot-assisted SIMO-OFDM systems. Telecommun. Syst..

[23] Raviteja P., Hong Y., Viterbo E. (2018). Millimeter Wave Analog Beamforming With Low Resolution Phase Shifters for Multiuser Uplink. IEEE Trans. Veh. Technol..

[24] Maneiro-Catoira R., Brégains J., García-Naya J.A., Castedo L. (2018). Analog Beamforming Using Time-Modulated Arrays With Digitally Preprocessed Rectangular Sequences. IEEE Antennas Wirel. Propag. Lett..

[25] Elnoubi S., Abdallah W. Minimum bit error rate (MBER) pre-FFT beamforming for OFDM communication systems. Proceedings of the 2012 Japan-Egypt Conference on Electronics, Communications and Computers (JEC-ECC).

[26] Tabata T., Fujimoto M., Hori S., Wada T., Asato H. Incoming waves separating adaptive array for ISDB-T mobile reception. Proceedings of the 2016 International Symposium on Antennas and Propagation (ISAP).

[27] Matsuoka H., Kasami H., Tsuruta M., Shoki H. A smart antenna with pre- and post-FFT hybrid domain beamforming for broadband OFDM system. Proceedings of the WCNC IEEE 2006 Wireless Communications and Networking Conference.

[28] Alihemmati R., Shishegar A.A., Hojjat N., Dadashzadeh G., Boghrati B., Mehrtash A. Comparison of the Smart Antenna Architectures for OFDM-WLAN Systems in a Rich Multipath Environment based on a Spatio-Temporal Channel Model. Proceedings of the PIMRC 2005 IEEE 16th International Symposium on, Personal, Indoor and Mobile Radio Communications.

[29] Zhang X., Feng B., Xu D. (2008). Blind joint symbol detection and DOA estimation for OFDM system with antenna array. Wirel. Pers. Commun..

[30] Hong Y.J. Vigorous Study on Pre-FFT Smart Antennas in OFDM. Proceedings of the 2011 Eighth International Conference on Information Technology: New Generations.

[31] Lei Z., Chin F. Post and pre-FFT beamforming in an OFDM system. Proceedings of the 2004 IEEE 59th Vehicular Technology Conference, 2004, VTC 2004-Spring.

[32] Kim C.K., Lee K., Cho Y.S. (2000). Adaptive beamforming algorithm for OFDM systems with antenna arrays. IEEE Trans. Consumer Electron..

[33] Seydnejad S., Akhzari M.S. CCI suppression and channel equalization in pilot-assisted OFDM systems by space-time beamforming. Proceedings of the 2011 International Conference on Communications and Signal Processing.

[34] Gökceli S., Uslu M., Kurt G.K., Özbek B., Alakoca H., Durmaz M.A. Implementation of pre-FFT beamforming in MIMO-OFDM. Proceedings of the 2015 9th International Conference on Electrical and Electronics Engineering (ELECO).

[35] Wu C.F., Chen C.H., Shiue M.T. Decision-Directed Beamforming and Channel Equalization Algorithm for IEEE 802.11n OFDM Systems. Proceedings of the 2016 International Symposium on Computer, Consumer and Control (IS3C).

[36] Alkhateeb A., Alex S., Varkey P., Li Y., Qu Q., Tujkovic D. (2018). Deep Learning Coordinated Beamforming for Highly-Mobile Millimeter Wave Systems. arXiv.

[37] Tsinos C.G., Ottersten B. (2018). An Efficient Algorithm for Unit-Modulus Quadratic Programs With Application in Beamforming for Wireless Sensor Networks. IEEE Signal Process. Lett..

[38] Gangaju S., Satyal S. (2015). Adaptive RLS Beamforming for MIMO-OFDM using VBLAST. Int. J. Comput. Appl..

